# Agreement between chest ultrasonography and chest X-ray in patients who have undergone thoracic surgery: preliminary results

**DOI:** 10.1186/s40248-019-0171-x

**Published:** 2019-03-04

**Authors:** Andrea Smargiassi, Riccardo Inchingolo, Marco Chiappetta, Leonardo Petracca Ciavarella, Stefania Lopatriello, Giuseppe Maria Corbo, Stefano Margaritora, Luca Richeldi

**Affiliations:** 1 Respiratory Medicine, Fondazione Policlinico Universitario A. Gemelli IRCCS, Rome, Italy; 2 Thoracic Surgery, Fondazione Policlinico Universitario A. Gemelli IRCCS, Rome, Italy; 3 Università Cattolica del Sacro Cuore, Rome, Italy

**Keywords:** Chest ultrasound, Innovative biotechnologies, Thoracic surgery, Chest X-ray, Ultrasonography

## Abstract

**Background:**

Chest Ultrasonography (chest US) has shown good sensibility in detecting pneumothorax, pleural effusions and peripheral consolidations and it can be performed bedside.

**Objectives:**

The aim of the study was to analyze agreement between chest US and chest X-ray in patients who have undergone thoracic surgery and discuss cases of discordance.

**Methods:**

Patients undergoing thoracic surgery were retrospectively selected. Patients underwent routinely Chest X-ray (CXR) during the first 48 h after surgery. Chest US have been routinely performed in all selected patients in the same date of CXR. Chest US operators were blind to both reports and images of CXR. Ultrasonographic findings regarding pneumothorax (PNX), subcutaneous emphysema (SCE), lung consolidations (LC), pleural effusions (PE) and hemi-diaphragm position were collected and compared to corresponding CXR findings. Inter-rater agreement between two techniques was determined by Cohen’s kappa-coefficient.

**Results:**

Twenty-four patients were selected. Inter-rater agreement showed a moderate magnitude for PNX (Cohen’s Kappa 0.5), a slight/fair magnitude for SCE (Cohen’s Kappa 0.21), a fair magnitude for PE (Cohen’s Kappa 0.39), no agreement for LCs (Cohen’s Kappa 0.06), high levels of agreement for position of hemi-diaphragm (Cohen’s Kappa 0.7).

**Conclusion:**

Analysis of agreement between chest X-ray and chest US showed that ultrasonography is able to detect important findings for surgeons. Limitations and advantages have been found for both chest X-ray and chest US. Knowing the limits of each one is important to really justify and optimize the use of ionizing radiations.

## Background

After thoracic surgery, surgeons need to monitor and manage clinical course of patients. Some important pathological findings are constantly searched and focused on to take decisions about chest tube removal, vacuum suction, antibiotic therapies, bronchoscopy etc.

Findings that are always evaluated are pneumothorax, subcutaneous emphysema, pleural effusions, lung consolidations and diaphragm displacement. Chest x-ray (CXR) during the first 48 h after surgery is the easiest diagnostic technique to perform in order to check these alterations although its use is controversial [1–3].

However, in the last years chest ultrasonography (Chest US) has been proposed as a bedside technique useful in many pathologic conditions. It has already been demonstrated its utility just for those conditions previously listed [4–7].

Aim of this paper is to study the level of agreement for those pathological conditions between chest ultrasonography and chest X-ray and to focus on cases of disagreement to identify advantages and limits of each technique. The final goal of this study is to understand whether chest US can limit, optimize and justify (not replace) ionizing radiations during the first 48 h after thoracic surgery and whether chest US can lead to rational use of chest X-ray.

## Materials and methods

### Study population

Study population ruled in patients admitted to Thoracic Surgical Department of University Hospital “Agostino Gemelli”, Rome, Italy, who have undergone to thoracic surgery either in open chest technique (lateral thoracotomy/mini-thoracotomy) or in uni/multi-portal VATS. Patients undergoing pneumonectomy have been ruled out because of ICU-admission after surgery. Patients were retrospectively selected in a 2-month sample period, from January to February 2017. In our structure, patients undergo routinely chest ultrasonography after thoracic surgery in the first 48 h. Selected patients have routinely undergone to both Chest ultrasonography and Chest X-Ray in the first 48 h after thoracic surgery. Patients have been included in this retrospective analysis when chest X-ray and chest ultrasonography have been performed one from the other in less than 3 h. No clinical changes had to be reported before performing the second technique.

### Chest ultrasonography

Ultrasonographic assessment was performed using MyLab™ 50 CV machine (Esaote, Genova, Italy) equipped with convex (2–5 MHz) and linear (7–13 MHz) probes.

All ultrasonographic evaluations were performed by pneumologists (AS and RI) and by a thoracic surgeon (MC) with a consolidated expertise in lung ultrasonography, blinded to Chest X-rays reports and images.

Each patient was asked initially to stay seated for dorsal sonographic scans, then to lie in a supine position for anterior and lateral scans. Monolateral ultrasonographic evaluation was performed according to surgical procedure. A bilateral assessment was performed only to compare sliding sign e position of hemi-diaphragm between right and left hemithorax.

The convex probe was used firstly to look for pleural effusions, lung consolidations, curtain sign and position of emidiaphagm. Then, the linear probe was used to detect sliding sign, pneumothorax (PNX), subcutaneous emphysema and pleural abnormalities. Images and videos were acquired and stored. A subsequent evaluation by 2 pneumologist and a thoracic surgeon (AS, RI, MC) with high expertise in lung ultrasonography was performed in order to collect and report in particular 5 ultrasonographic findings:

1. Pneumothorax (PNX): it is described by the detection of lung points, the focal absence of sliding of the pleural line and the absence of sonographic interstitial syndrome [5].
2. Subcutaneous Emphysema (SCE): it is characterized by the presence of air under the skin in the layers of the chest wall. Air is able to hamper the ultrasound beam to go beyond tissues till to the pleural line. It is easily detected by ultrasounds but it can hide ultrasonographic findings beyond the air barrier. It has a characteristic crackling feel to the touch [6].
3. Pleural Effusion (PE): it is described as hypo-anechoic fluid collection, bordered by the parietal pleural layer on the surface and by the visceral layer in its depth. Free-flowing pleural effusions lay in lower areas for gravitational effects. Instead, loculated pleural effusions appear as well-defined fluid structure [5, 8, 9].
4. Lung consolidations (LC): In chest ultrasonography, they appear as subpleural hypoechoic solid structures that are multiform in shape and dimensions. Usually well delimitated, LC can be surrounded by focal sonographic interstitial syndrome. This pattern is described as alterations of the pleural line with merged vertical artifacts and B-lines, which is typically indicative of a pre-consolidated state of the lung [7].
5. Position of hemi-diaphragm: using a convex probe it is possible to detect the curtain sign described as the border between the artifactual field of expanded lung and the morphological field of parenchymal organs in abdomen. Curtain sign is indicative of the position of hemi-diaphragm when compared with the position of the contralateral. Whether a free-flowing PE or a LC are detected, curtain sign is not identifiable. In this case hemidiaphragm is identified by using both convex and linear probe. It appears as a thin three-layer muscle lying between parenchymal organs in abdomen and either LC or PE or both [5, 10, 11].

After having revised Chest US, operators declared if it would have been useful to perform Chest X-ray for a panoramic view or if chest US findings could be sufficient to get the most relevant information for the surgeon after surgery.

### Chest X-ray

All patients systematically undergo AP-projection chest X-ray in inspiration during the first 48 h after surgery. Chest X-ray was required to get relevant information for surgeons in order to take medical subsequent decisions (e.g. removal of chest tube drainage, connection to vacuum, bronchoscopy).

Reports and images have been obtained in order to collect the same findings listed for chest ultrasonography: pneumothorax, subcutaneous emphysema, pleural effusion, lung consolidation, and position of hemi-diaphragm compared with contralateral.

### Statistical analysis

A descriptive analysis was reported by computing mean values and standard deviations. Inter-rater agreement between chest US and chest X-ray for the 5 listed findings was determined by Cohen’s kappa-coefficient statistic. Inter-rater agreement magnitude has been considered based on Landis and Koch proposal: Cohen’s kappa values < 0 as indicating no agreement; 0–0.20 as slight, 0.21–0.40 as fair, 0.41–0.60 as moderate, 0.61–0.80 as substantial, and 0.81–1 as almost perfect agreement. Simple percentage agreement was also reported.

No comparison with gold standard test (chest CT scan) was available because it was not clinically and ethically required for this study population.

## Results

The study population consisted of 24 patients (15 males) with an average age of 63.6 ± 15.5 years. Fourteen patients have undergone to uni-portal VATS, 2 patients to multi-portal VATS, 5 patients to thoracotomy, 1 case of mini-thoracotomy and 2 cases of robotic thymectomy.

Chest ultrasonography was performed 32,8 ± 8,8 h after surgery. Chest US was able to detect 20 PNX (in all cases lung point was identified), 16 cases of subcutaneous emphysema, 18 pleural effusions, 15 lung consolidations and 5 cases of elevated hemidiaphragm compared with contralateral (Table 1).

**Table 1:** Chest US findings

Chest US findings	
Pneumothorax	20
Subcutaneous Emphysema	16
Pleural effusion	18
Lung consolidation	15
Elevated Hemidiaphragm	5

Chest X-ray revealed 15 PNX, 10 SCE, 13 PE, 15 LC, 3 elevated hemidiaphragm (Table 2).

**Table 2:** Chest X-ray findings

Chest X-ray findings	
Pneumothorax	15
Subcutaneous Emphysema	10
Pleural effusion	13
Lung consolidation	15
Elevated Hemidiaphragm	3

Both chest Us and chest X-ray could detect contemporaneously more than one finding in the same patients.

Agreement study between the two techniques was performed for each finding.

As far as PNX is concerned, percentage of agreement was 79% with Cohen’s Kappa 0.5, resulting in a moderate magnitude of inter-rater agreement. Both chest US and chest X-ray detected PNX in 15 cases. Both techniques didn’t detect it in 4 cases. Discordance in 5 cases (Table 3).

**Table 3:** Agreement for Pneumothorax (PNX)

PNX	Chest X-ray	
Chest US	Negative	Positive
Negative	4	0
Positive	5	15

Cohen’s Kappa: 0.5

Percentage of agreement: 79%

SCE has shown 58% of agreement with Cohen’s Kappa 0.21, indicating a slight/fair magnitude. In this case, both techniques were in agreement both to identify SCE in 8 subjects and to not identify it in 6 subjects. Discordance in 10 cases (Table 4).

**Table 4:** Agreement for Subcutaneous Emphysema (SCE)

SCE	Chest X-ray	
Chest US	Negative	Positive
Negative	6	2
Positive	8	8

Cohen’s Kappa: 0.21

Percentage of agreement: 58%

As to PE, agreement was 70% and Cohen’s Kappa 0.39, showing a fair magnitude. In 12 cases, PE was positively detected by both techniques. In 5 cases PE was concordantly absent. Discordance has been reported for 7 cases (Table 5).

**Table 5:** Agreement for Pleural effusion (PE)

PE	Chest X-ray	
Chest US	Negative	Positive
Negative	5	1
Positive	6	12

Cohen’s Kappa: 0.39

Percentage of agreement: 70%

For LC, instead, no agreement has been found (Cohen’s Kappa 0.06) with percentage of concordance of 50%. Disagreement was reported therefore in 12 out of 24 patients (Table 6).

**Table 6:** Agreement for Lung consolidation (LC)

LC	Chest X-ray	
Chest US	Negative	Positive
Negative	3	6
Positive	6	9

Cohen’s Kappa: 0.06

Percentage of agreement: 50%

Finally, diaphragmatic displacement has shown high levels of concordance (91%) with substantial inter-rater agreement (Cohen’s Kappa 0.7). Normal position in 18 cases and agreement on elevation in 3 subjects. Only 2 cases of disagreement were reported (Table 7).

**Table 7:** Agreement for Diaphragm Displacement (DD)

DD	Chest X-ray	
Chest US	Negative	Positive
Negative	18	0
Positive	2	3

Cohen’s Kappa: 0.70

Percentage of agreement: 91%

## Discussion

Level of agreement between chest X-ray and chest US has been computed for 5 pathological findings that are important for surgeons managing clinical course after surgery. Cases of discordance are discussed below for each finding in order to identify advantages and limits of each technique.

As to PNX, chest US and Chest X-ray showed a moderate inter-rater agreement. Discussion deserves for case of discordance. In 5 cases Chest Us was positive for PNX and chest X-ray negative. No cases were positive for Chest X-ray and negative for Chest US. In all 5 cases Chest US identified PNX by detecting lung point(s).

Lung point has been reported in literature with high specificity for PNX [12, 13]. When detected, PNX is always present. Thus, we can conclude that chest US has higher sensitivity for PNX, as already reported in literature, if compared with chest X-ray. It has been reported that Chest X-ray has good specificity for PNX, but it has no high levels of sensitivity [14], especially when performed in one AP-projection and in supine position [15, 16]. However, although chest US can be considered more specific and more sensible than X-ray, a limitation has to be discussed: US is not useful to correctly estimate the extension of PNX. We can suspect big amount of PNX when lung point is detected, in clinostatic position, far from the parasternal line of the thorax and near to axillary lines. In these cases, performing chest X-ray is mandatory in order to correctly estimate extension [17].

Instead, when chest X-ray is not able to detect PNX, we can believe that it has minimal extension without clinical significance after surgery. The importance to detect even minimal amount of PNX could be useful; however in some circumstances like persistent air leaks after surgery or in case of necessity for mechanical ventilation or CPAP. To summarize, we believe that chest US might be useful both to detect even minimal amount of PNX with high sensitivity and specificity, and to correctly optimize the use of ionizing radiations when required.

Subcutaneous emphysema showed a slight/fair inter-rater agreement between the two techniques. Ten cases of discordance need to be discussed. In 8 cases out of these 10, chest US was positive for SCE and chest X-ray was negative. Chest US is very sensitive for air leaks in the tissue layers of the chest wall. Air is able to hamper the ultrasounds to go beyond tissues. Thus, SCE is easily detected, also minimal amounts, when ultrasounds are not able to reach the pleural line and are reflected while crossing tissues [5].

Hence, it is not surprising whether chest US has been able to detect more SCE than chest X-ray. However, small amounts of SCE are not clinically relevant.

**Fig. 1:**
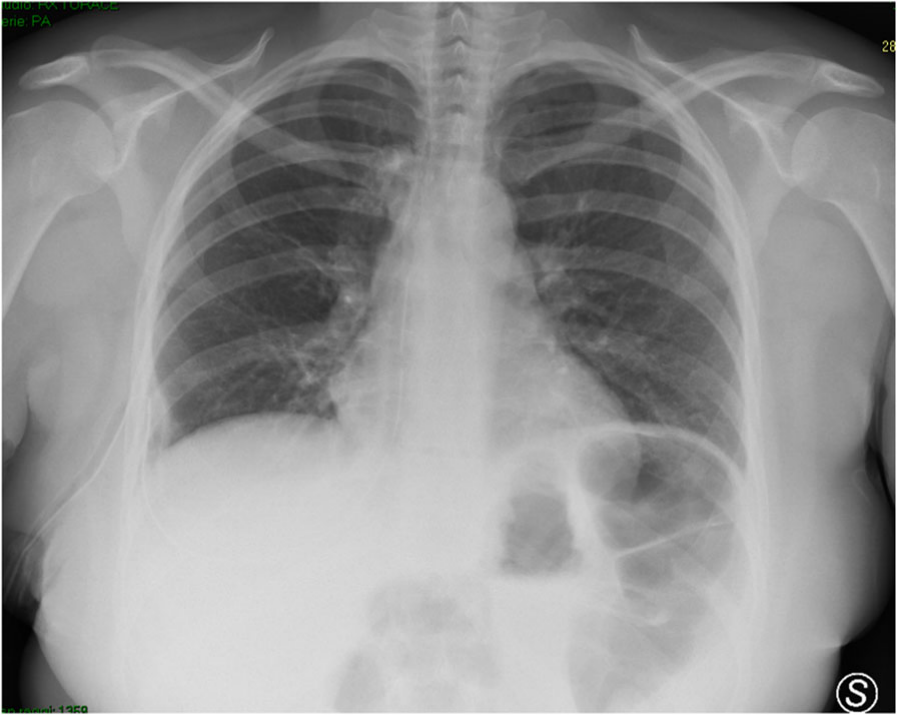
A case of subcoutaneous emphysema localized only around the surgical wound. It has been missed by chest US because of local medications

In 2 cases out of 10, instead, chest X-ray reported SCE and chest US was negative. In these two cases, SCE, not clinically relevant, was focally localized around the surgical wound. Chest US wasn’t able to detect SCE because of local medication with gauze and patch that had hampered local examination (Fig. 1).

Also for SCE we believe that chest US could be useful to better understand extension and clinical importance. The limit of US examination in these cases is that extensive SCE diffusely hampers the ultrasound beam to asses pleural and lung findings. Chest US examination is limited when SCE is diffuse and chest X-ray is mandatory.

**Fig. 2:**
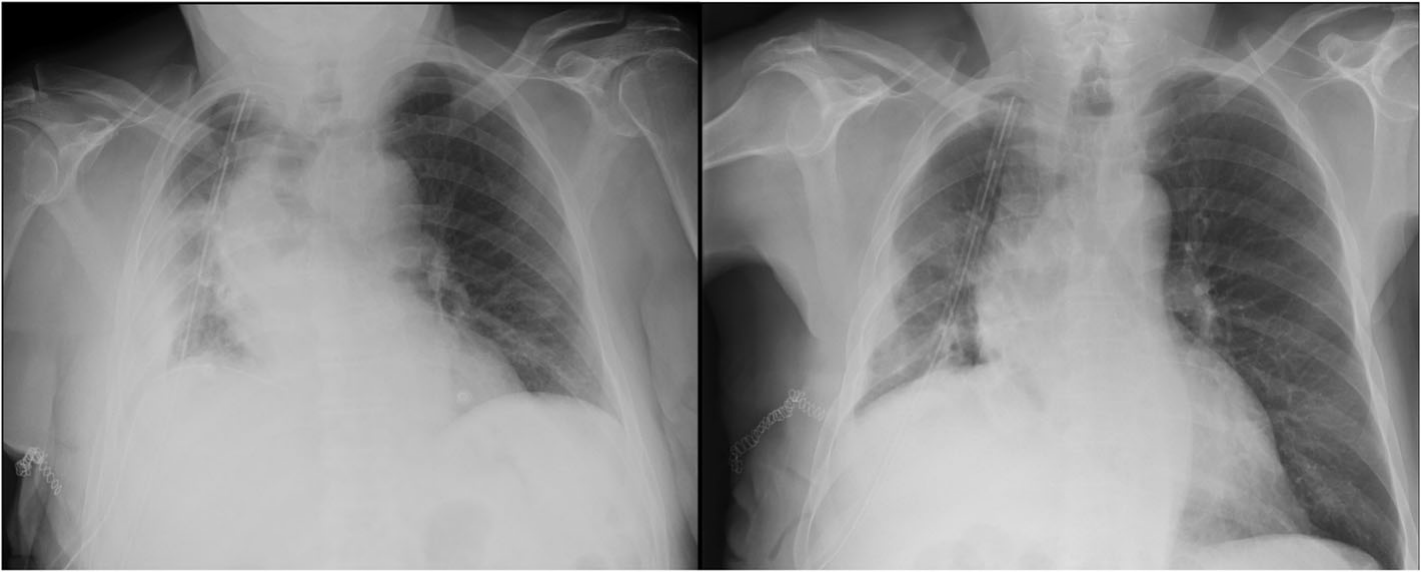
Chest x-ray performed before (on the right) and after (on the left) bronchoscopy. Chest x-ray reported the presence of loculated pleural effusion, which was correctly identified as obstructive atelectasis by Chest US (see Fig. 4)

For PE, it is known that Chest US can represent the gold-standard technique [18–20]. Inter-rater agreement with Chest X-ray showed a fair magnitude. Seven cases of discordance have been reported. It is not surprising that Chest US was able to identify 6 cases that chest X-ray missed. Pleural effusions, also minimal amounts, are well detected by ultrasounds. Only in one case chest X-ray reported loculated PE in disagreement with US. In this case, chest US was confident for lung consolidation, for instance obstructive atelectasis. The patient underwent multiple bronchoscopies to remove obstruction by secretions. Figure 2 reported chest X-ray performed pre- and post-bronchoscopies. In our sample chest US confirmed to be referral technique for PE as already reported in literature.

As far as LCs are concerned, chest X ray and chest US showed the worst level of agreement. In 6 cases chest X-ray reported consolidations that chest US wasn’t able to find. All 6 cases have been described as central consolidations compatible with lung contusions and atelectasis as result of surgery.

Central consolidations are not assessable by ultrasounds because the ultrasound beam is not able to explore inflated lung parenchyma [4, 7]. The condition to become detectable by ultrasounds is that the consolidation has to reach and touch visceral pleura. In these cases, ultrasounds are able to detect even small consolidations, difficult to be seen by chest X-ray [21, 22]. This is the reason why in other 6 cases chest US reported consolidations that weren’t missed by one AP-projection Chest X-ray.

However, it is important to say that lung contusions after surgery are frequent and not clinically relevant. It is important, instead, to detect flogistic LCs and pneumonias in that period. It has already been reported in literature that chest US has good sensitivities for pneumonias, better than chest X-ray [21, 22].

The limit for chest US after surgery for detecting pneumonias could be represented by the presence of large pneumothorax or massive SCE that hampers ultrasonographic assessment. As already discussed before, however, in case of either suspected large pneumothorax or massive SCE, chest X-ray is mandatory.

**Fig. 3:**
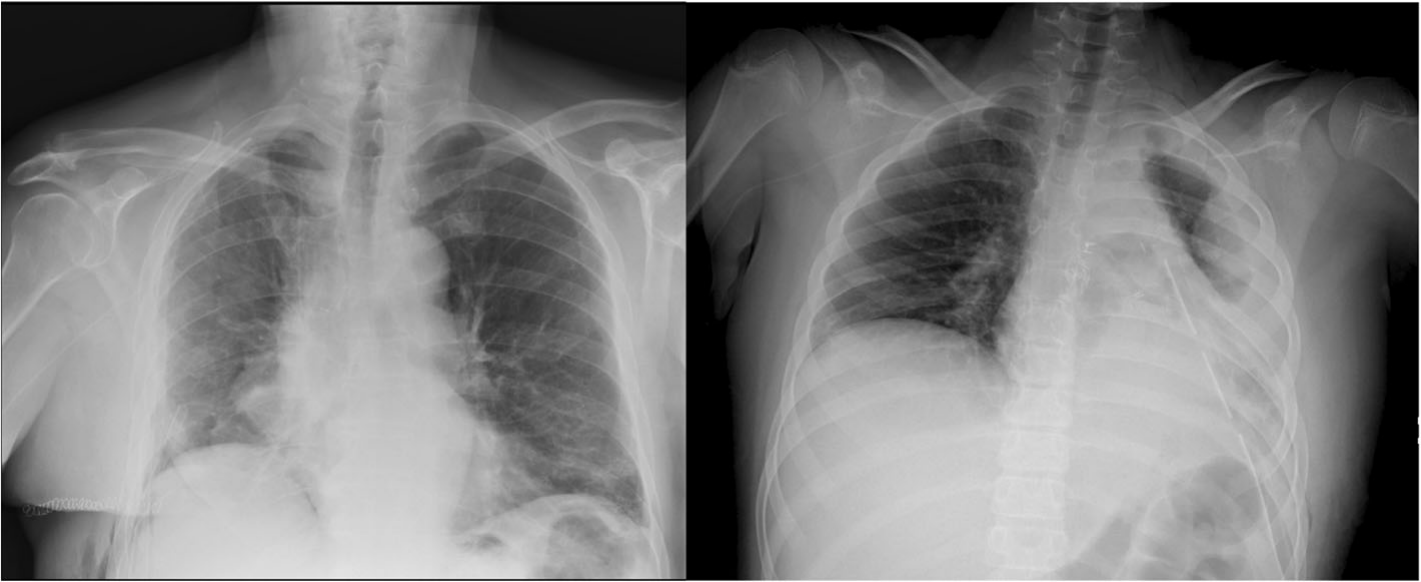
On the right: a case of elevated hemidiaphragm detected by both chest US and chest X-ray. On the left: Elevated left hemidiagram not easily detectble by chest x-ray because of concomitant pleural effusion and basal lung consolidation

Finally, position of hemi-diaphragm showed significant inter-rater agreement between the two techniques. Only 2 cases of discordance have been reported. One case of elevated hemidiaphragm on the left and one case on the right. In both cases chest US revealed an elevated hemi-diaphragm which was missed by one AP-projection Chest X-ray. Chest X-ray wasn’t able to clearly detect the position of hemi-diaphragm because of the presence of pleural effusion and basal lung consolidation that covered diaphragm displacement. (Fig. 3).

**Fig. 4:**
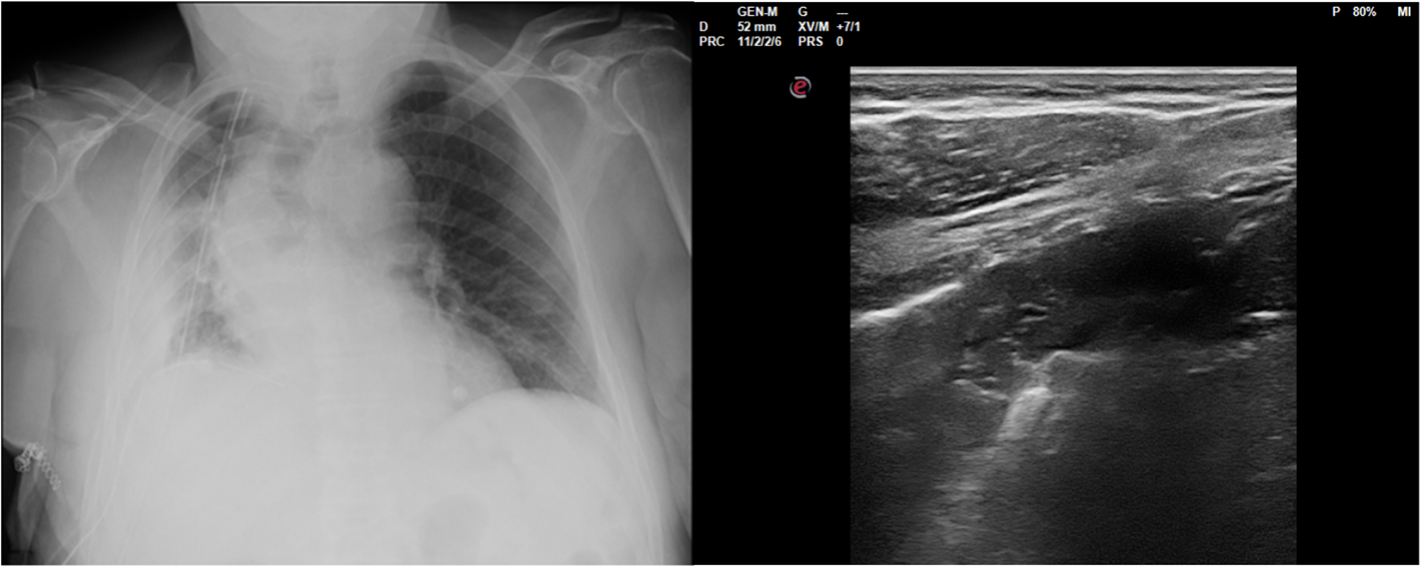
The same case of Fig. 2. Chest US reported LC compatible with obstructive atelectasis and reduction of volume of the hemithorax. Chest X-ray was required for a panoramic view

After echographic assessment, in 5 cases out of 24, it would have been useful to perform chest x-ray. Three cases of massive SCE, one case of obstructive atelectasis with reduction of volume of the hemithorax for a panoramic view (Fig. 4) and one case of hydro-pneumothorax.

## Conclusion

During the first 48 h after thoracic surgery, surgeons usually need chest X-ray to check some important findings that have to be related to clinical conditions. Analysis of agreement between chest X-ray and chest US showed that ultrasonography, easily performed bedside, is able to detect important findings for surgeons. Some limitations have been found and discussed for both chest X-ray and chest US. However, knowing the limits of chest US is important to really justify and optimize the use of chest X-ray in some cases. There is no competition between these two techniques, but it must be the physician that has to choose the better one, knowing advantages and limitations of each one, case by case. In this optimized diagnostic path, chest US can limit the daily use and justify rational use of CXR. [23, 24]. This retrospective pilot analysis could represent the first step towards the development of a prospective study on the usefulness of chest ultrasound after thoracic surgery.

## Abbreviations

chest US: Chest Ultrasonography
CXR: Chest X-ray
LC: Lung Consolidations
PE: Pleural Effusions
PNX: Pneumothorax
SCE: Subcutaneous Emphysema
